# Treatment of Sternoclavicular Joint Osteomyelitis with Debridement and Delayed Resection with Muscle Flap Coverage Improves Outcomes

**DOI:** 10.1155/2014/747315

**Published:** 2014-03-12

**Authors:** Jason L. Muesse, Shanda H. Blackmon, Warren A. Ellsworth, Min P. Kim

**Affiliations:** ^1^Department of Surgery, Houston Methodist Hospital, 6550 Fannin Street, SM 1661, Houston, TX 77030, USA; ^2^Department of Surgery, Weill Cornell Medical College, Houston Methodist Hospital, 6550 Fannin Street, SM 1661, Houston, TX 77030, USA; ^3^The Institute for Reconstructive Surgery, Houston Methodist Hospital and Division of Plastic Surgery, Baylor College of Medicine, Houston, TX 77030, USA

## Abstract

The objective of this study was to evaluate the efficacy of various treatment options for sternoclavicular joint osteomyelitis. We evaluated patients with a diagnosis of sternoclavicular joint osteomyelitis, treated at our hospital from 2002 to 2012. Four treatment options were compared. Three out of twelve patients were successfully cured with antibiotics alone (25%). Debridement with or without negative pressure therapy was successful for one of three patients (33%). Simultaneous debridement, bone resection, and muscle flap coverage of the acquired defect successfully treated one of two patients (50%). Debridement with delayed bone resection and muscle flap coverage was successful in five of five patients (100%). Osteomyelitis of the sternoclavicular joint is a rare disease that has become more prevalent in recent years and can be associated with increasing use of long-term indwelling catheters. Initial debridement with delayed bone resection and pectoralis major muscle flap coverage can effectively treat sternoclavicular joint osteomyelitis.

## 1. Introduction

Sternoclavicular joint (SCJ) osteomyelitis is an infection of the joint where the clavicle attaches to the manubrium and is usually associated with an abscess in the area. It is a very rare condition with approximately 225 cases reported in the past 45 years [1–21]. All of the patients reported in the literature were treated with antibiotics initially and some patients underwent surgical management of sternoclavicular joint osteomyelitis when symptoms did not improve on antibiotics. Previously described surgical techniques include simple incision with debridement and drainage with or without negative pressure dressing [2, 3, 22–24], resection of the sternoclavicular joint with healing by secondary intention [3, 25], and resection with simultaneous flap coverage using pectoralis major, latissimus dorsi, or rectus abdominus muscles [2, 22–24, 26]. Patients who underwent simple incision with debridement and drainage either have prolonged open wound care with median of 12 weeks [2] or a high failure rate up to 80% [23]. Patients who underwent resection with immediate pectoralis major muscle flap had wound complication rates up to 50% [2].

Our experience in treating this condition and evaluating limitations of previously described techniques for management of sternoclavicular joint osteomyelitis has led to the development of a novel surgical strategy for treatment. We propose initial incision and debridement of the infected sternoclavicular joint followed by delayed resection and pectoralis major muscle flap advancement as the optimal treatment for patients diagnosed with sternoclavicular joint osteomyelitis requiring surgery.

## 2. Materials and Methods

**Figure 1:**
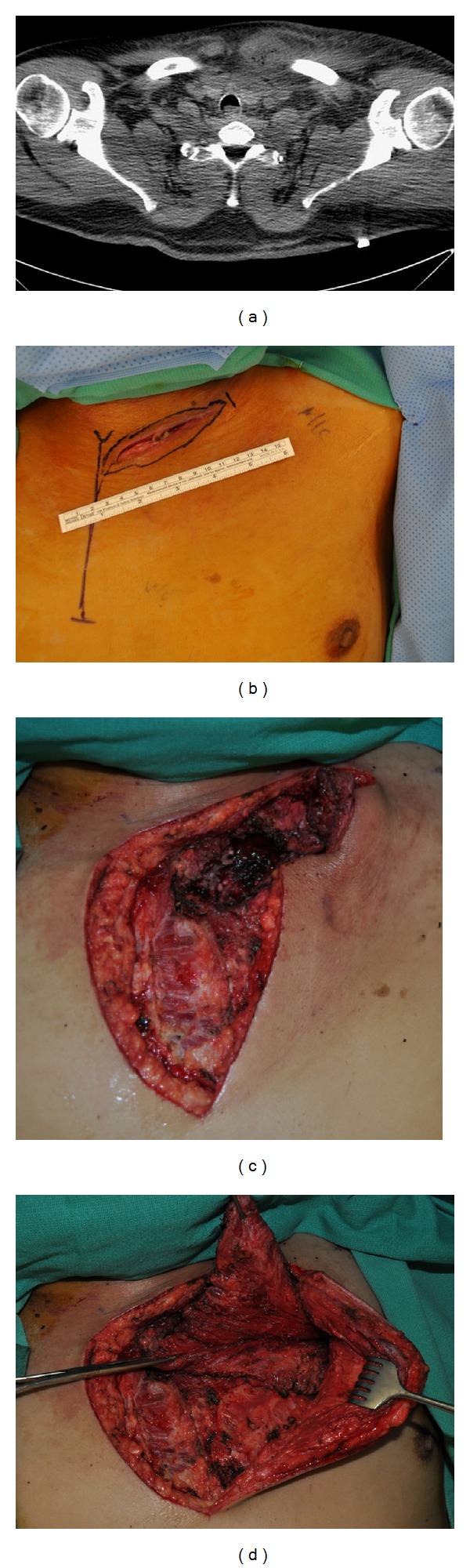
(a) Computerized Tomography scan demonstrating left sternoclavicular joint abscess and osteomyelitis in 57-year-old male with a history of infected tunneled hemodialysis catheter (removed). (b) Photograph demonstrating wound 3 weeks after incision and debridement, before resection of infected bony structures. (c) Photograph demonstrating surgically acquired defect of chest wall following resection of infected bony structures. (d) Photograph demonstrating mobilization of left pectoralis major muscle flap being advanced into surgically acquired defect of chest wall.

Our study was approved by the institutional review board at Houston Methodist Hospital Research Institute. We searched the administrative database at Houston Methodist Hospital for admissions from January 1, 2002, to June 1, 2012, with the diagnosis of osteomyelitis of the shoulder region (ICD-9 Code 730.21) or osteomyelitis of unspecified location (ICD-9 Code 730.20) as there is no ICD-9 code for osteomyelitis of the sternoclavicular joint. Our inclusion criteria were the diagnosis of osteomyelitis of the sternoclavicular joint based on radiographic findings (Figure 1(a)) or findings of sternoclavicular osteomyelitis at the time of initial debridement. We excluded patients who did not have diagnostic tests or treatments performed at our institution. We collected information from patient charts about the date of diagnosis, etiology of infection, organism responsible for infection, and treatment course.

Four treatment options were compared for efficacy. We evaluated (1) antibiotics alone, (2) incision and debridement with bone resection alone as definitive therapy, (3) incision and debridement with bone resection with immediate muscle flap advancement, and (4) incision and debridement with delayed bone resection and muscle flap advancement. We considered success of treatment to be resolution of infection without the requirement for further therapy. If patients failed one treatment option and went on to another therapy, their success or failure in each involved modality contributed to the determination of the overall efficacy of that modality.

### 2.1. Preoperative Imaging

Imaging of the chest and lower neck with either CT or MRI was obtained based on clinical suspicion of SCJ infection [1, 2, 10]. Obtaining the study with intravenous contrast use can aid in enhancing the abscess and confirming patency of the arteries that will provide blood flow to the anticipated muscle flap. On imaging, the abscess cavity may or may not contain gas bubbles, and often very high-density fluid within the abscess cavity may track into the neck region or mediastinum.

### 2.2. Surgical Technique

The strategy that we advocate for the treatment of sternoclavicular joint osteomyelitis involves incision and debridement with or without negative pressure therapy for two to three weeks, followed by bone resection and pectoralis major muscle flap advancement.

During the initial debridement, an incision was made over the sternoclavicular joint from the manubrium to the midclavicle. The skin above the abscess cavity was entered sharply and drained with Yankauer suction followed by pulse lavage. The incision usually averaged five to eight centimeters in length based on the degree of abscess cavity found on the preoperative imaging and was adequate enough to ensure drainage. Aerobic and anaerobic cultures of abscess fluid were obtained. Segments of infected appearing sternoclavicular joint, manubrium, and/or clavicle were biopsied with Rongeurs and sent for culture and pathologic evaluation to confirm osteomyelitis and rule out malignancy, but no definitive bone resection was carried out at this time. Some patients underwent negative pressure therapy with Wound V.A.C. (KCI, San Antonio, TX, USA) placement at the time of the initial incision and drainage while other patients who had a copious amount of purulence in their wound were treated initially with wet to dry gauze for a few days. Once there was minimal drainage and the character of the drainage was serous, a Wound V.A.C. was placed at bedside for temporary closure until the patient underwent definitive bone resection and pectoralis major flap advancement. The patient was then discharged home or to an assisted care facility with IV antibiotics and home health care to assist with Wound V.A.C. therapy dressing changes three times per week. The patient was scheduled for definitive bone resection and muscle flap coverage two to three weeks from the date of the original operation.

During the second operation, the plastic surgeon started the case by removing the negative therapy pressure device if present (Figure 1(b)) and extending the inverted “L” shaped incision laterally and caudally. The previous drainage incision was excised. The ipsilateral pectoralis major muscle was then released from its chest wall attachments. From this incision, lateral attachments of the pectoralis major muscle to the humerus could be reached and released if more length was needed to cover the defect. If necessary, additional length could be gained by performing a counter incision in the axilla to free the lateral attachments of the pectoralis major to the humerus. Care must be taken to not disrupt the vascular pedicle, pectoral branch of the thoracoacromial artery, of the pectoralis major muscle flap during debridement. With the pectoralis major muscle flap mobilized, the thoracic surgeon then resected any infected or necrotic bone (Figure 1(c)). With the purulent fluid drained from the area and inflammation decreased from the initial operation, viability of the bone structures could be more easily assessed. Bone resection was performed until healthy appearing bone was encountered. The plastic surgeon then completed the operation by advancing the pectoralis major muscle into the acquired defect and suturing it loosely to the underlying pectoral fascia with absorbable suture (Figure 1(d)). Closed suction drains were always inserted by the plastic surgeon above and below the pectoralis major muscle flap and the patient was discharged with these drains in place. Overlying dermis was closed in layers and skin closed with vertical mattress nylon sutures or skin staples. Drains were removed by the plastic surgeon when output was less than 30 cc in 24-hour period. At follow-up appointments, the range of motion was also assessed. Physical and occupational therapy was encouraged postoperatively.

## 3. Results

Our database search based on ICD coding yielded 328 admissions from 260 patients. Twelve patients with osteomyelitis of the SCJ met our inclusion and exclusion criteria. Average age at presentation was 58 ± 11 years old with mostly Caucasian (*n* = 7, 58%) and male (*n* = 8, 67%) patients. Most common comorbidities were hypertension (*n* = 8, 73%), BMI > 30 (*n* = 7, 64%), diabetes mellitus (*n* = 6, 50%), and history of tobacco abuse (*n* = 5, 45%, Table 1). Eight of 12 patients (67%) presented in the past four years (2008 to 2012), two patients (17%) presented between 2001 and 2003 of the study, and two patients (17%) presented between 2004 and 2007. All patients had physical symptoms, which prompted initial evaluation, including fever, pain with ipsilateral arm movement, swelling of ipsilateral arm or neck, and redness or warmth over the clavicle or neck region. Seven of 12 patients (58%) were associated with long-term central venous catheter use. Catheter types implicated as the infectious source included dialysis catheters, implanted and tunneled ports for long-term central venous access, and peripherally inserted central catheters (PICC lines). Other causes for the source of infection were infection at another noncatheter related site (Table 1). The organism most often implicated in the infection was* Staphylococcus aureus* (*n* = 8, 67%), followed by* Pseudomonas aeruginosa* (*n* = 2, 17%), Group B* Streptococcus* (*n* = 1, 8%), and* E. coli* (*n* = 1, 8%, Table 1).

Every patient was treated with antibiotics and three of the patients were successfully cured with antibiotics alone (25%, Table 2). Three patients underwent debridement of the affected area with definitive bone resection and no further plans for intervention, but only one of these three patients was treated successfully with this technique (33%). One of the two patients who failed went on to hospice care because of associated comorbidities and the other patient required further surgical intervention. A third treatment group, comprised of two patients, underwent debridement, resection of infected bone, and immediate muscle flap coverage of the acquired defect using a pectoralis major muscle flap. One of two patients was successfully treated with this method (50%) and the other patient developed a postoperative hematoma that required surgical drainage. A fourth group underwent initial debridement with delayed bone resection and pedicled ipsilateral pectoralis major muscle flap coverage two to three weeks later when initial inflammation had decreased and purulent drainage was at a minimum. Five patients were included in this group and none of these patients developed wound complications and all of the patients resolved the osteomyelitis of the sternoclavicular joint (100%). We were able to obtain excellent postoperative functioning with near complete range of motion. While a visible defect in the breast can be appreciated when disrobed, overall distortion of chest wall symmetry was minimal. Female patients had some mild distortion of breast architecture but did not report any dissatisfaction with asymmetry.

## 4. Discussion

Sternoclavicular joint osteomyelitis is a rare disease that has increased in incidence at our institution. From our review, a minority of patients were successfully treated with antibiotics alone with or without percutaneous drainage without debridement. These patients likely had very low grade infection without large abscess or osteomyelitis. However, based on our experience and published data, most patients fail this method of therapy. Many of the patients we have seen with SCJ infection had been on antibiotic therapy for weeks before we were consulted, with little or no improvement in symptoms.

Previously described surgical techniques include incision and drainage with drainage with simultaneous muscle flap coverage or simple incision and debridement with healing by secondary intention. A recent review of these two surgical approaches for treatment of SCJ infection in 20 patients showed problems with each method. There was a 50% complication rate with immediate flap closure including hematoma, dehiscence, and seroma. On the other hand, the patients who underwent open wound care required therapy for a median of 12 weeks [2]. We had similar finding in our small series in patients treated with these two methods. We had 50% wound complication with resection and immediate flap and prolonged wound care when no muscle flap was used. The debridement and open wound care had a very low success rate in another surgical series which showed that this approach was only successful in one out of six patients with the other five patients requiring additional procedures [23]. Open wound treatment in this patient population who often suffer from medical comorbidities results in prolonged length of time to healing and inconvenience to the patient, poor cosmetic result, increased costs, and possible introduction or worsening of osteomyelitis when bone is left exposed for long periods of time.

**Figure 2:**
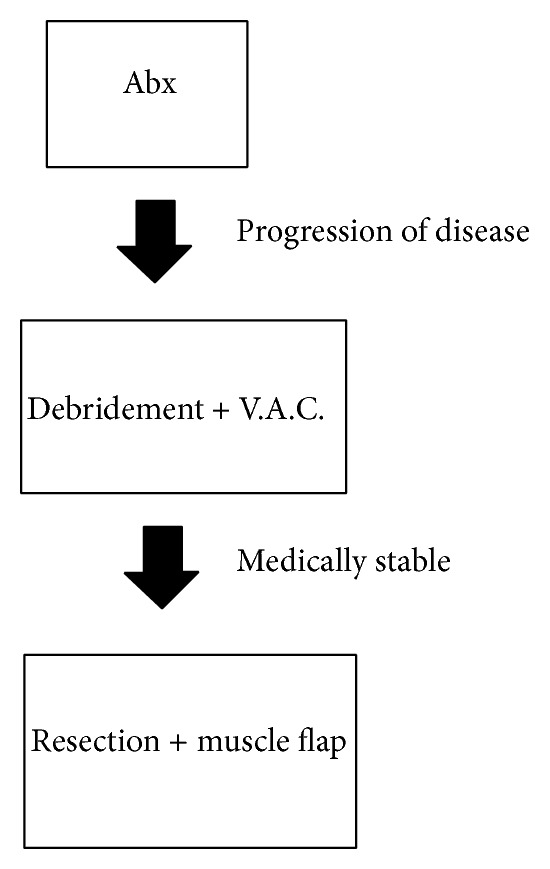
Algorithm of treatment of sternoclavicular joint osteomyelitis. Patients should be initially treated with systemic antibiotics. When there is progression of disease despite antibiotic treatment, the patient should undergo initial debridement and subsequent negative pressure therapy. If the patient is medically stable, then after two to three weeks the patient should have formal resection and muscle flap coverage.

Based on our early experience and review of the literature, we developed a new sequence of treating sternoclavicular joint osteomyelitis with incision and debridement followed by delayed bone resection and muscle flap coverage (Figure 2). Thus far, we have had a high success rate with each of five patients recovering from this sequence of operations without wound complication or recurrence. It appears to be superior to immediate bone resection and flap coverage for treatment of this condition. The time of open wound care of was approximately 3 weeks between the first and second operations, which is significantly less than the previously described median length of wound care of 12 weeks for open drainage alone.

We feel that our novel approach provides several advantages over other methods of treating this disease. With delayed bone resection and flap coverage, we had none of the previously decreased wound healing complications like seroma or hematoma or surgical site infections described with immediate bone resection and flap coverage. We feel that the viability of bony structures can be more easily assessed at the time of the second operation when purulent material has been adequately drained and inflammation has decreased. Also, delayed resection allows time for culture results and sensitivities from the initial debridement to return so that antibiotic therapy can be appropriately targeted in the interim if the offending microorganism is not known preoperatively. This prevents wound closure in the setting of the infected field if the patient is on inappropriate antibiotics, predisposing the patient to wound complications. Although not proven, we strongly feel that the vascularized muscle flap itself provides the additional benefit of direct antibiotic delivery to the site of infection once the flap is in place, which helps to eradicate the remaining microorganisms and obliterate dead space. Also, delaying bone resection and flap coverage allows time for pathologic confirmation that the affected bone is free from malignancy, which could alter surgical plans.

Our conclusions are limited by the small sample size of our case series, the lack of blinding, and the lack of randomization. However, since this is a very rare disease and there is no clear optimal management method reported in the literature, we believe that our series provides a novel experience treating this group of patients.

Based on the data generated in this study, we plan to treat all future patients who need surgical debridement for osteomyelitis of the sternoclavicular joint with initial incision and debridement followed by two to three weeks of wound care along with antibiotic treatment followed by delayed resection of infected bone and pectoralis major muscle flap advancement into the acquired defect. We believe that this is the best management for patients who need surgical intervention for sternoclavicular joint osteomyelitis.

## Figures and Tables

**Table 1:** Patient and sternoclavicular joint osteomyelitis characteristics.

	*n* = 12
Age, (y) mean ± SD	58 ± 11
Male, *n* (%)	8 (67)
Ethnicity, *n* (%)	
Caucasian	7 (58)
African American	1 (8)
Hispanic	4 (33)

	*n* = 11

Comorbidities, *n* (%)	
HTN	8 (73)
BMI > 30	7 (64)
DM	6 (55)
Smoker	5 (45)
CAD	4 (36)
Sleep apnea	4 (36)
Hyperlipidemia	4 (36)
Cancer	3 (27)
ESRD	2 (18)
CVA	2 (18)

	*n* = 12

Cause, *n* (%)	
Catheter	7 (58)
Infection at distant site	1 (8)
Skin biopsy	1 (8)
Unknown	3 (25)

	*n* = 12

Organism, *n* (%)	
*Staphylococcus aureus *	8 (67)
*Pseudomonas aeruginosa *	2 (17)
Group B* Streptococcus *	1 (8)
*E. coli *	1 (8)

BMIL: body mass index; CAD: coronary artery disease; CVA: history of cerebrovascular accident (stroke); DM: diabetes mellitus; ESRD: end-stage renal disease; HTN: hypertension; *n*: number of patients; SD: standard deviation.

**Table 2:** Efficacy of treatment.

	Success rate *n* (%)
Abx (*n* = 12)	3 (25)
I & D (*n* = 3)	1 (33)
Imm flap (*n* = 2)	1 (50)
Delay flap (*n* = 5)	5 (100)

Abx: antibiotics alone; Delay flap: incision and debridement with delayed bone resection and muscle flap advancement; I & D: incision and debridement, Imm flap: incision and debridement with bone resection and immediate muscle flap advancement.
